# Association between family members of dialysis patients and chronic kidney disease: a multicenter study in China

**DOI:** 10.1186/1471-2369-14-19

**Published:** 2013-01-18

**Authors:** Xianglei Kong, Li Liu, Li Zuo, Ping Yuan, Zhongxin Li, Wenge Li, Meishun Cai, Xiangmei Chen, Aili Jiang, Gang Long, Jinsheng Xu, Hongli Lin, Shixiang Wang, Wen Huang, Yiping Wang, Yidan Guo, Po Cao, Hua Wu, Qiang Jia, Luxia Zhang, Mei Wang, Haiyan Wang

**Affiliations:** 1 Renal Division, Department of Medicine, Peking University First Hospital; Peking University Institute of Nephrology; Key Laboratory of Renal Disease, Ministry of Health of China; Key Laboratory of Chronic Kidney Disease Prevention and Treatment (Peking University), Ministry of Education, 8 Xishiku Street, Xicheng District, Beijing, 100034, China; 2 Department of Nephrology, Qianfoshan Hospital, Shandong University, Jinan, China; 3 Dialysis and Transplantation Centers, The Third Central Hospital, Tianjin, China; 4 Department of Nephrology, Beijing ChaoYang Hospital, Beijing, China; 5 Department of Nephrology, China-Japan Friendship Hospital, Beijing, China; 6 Department of Nephrology, Peking University People’s Hospital, Beijing, China; 7 Department of Nephrology, The General Hospital of Chinese People’s Liberation Army, Beijing, China; 8 Department of Nephrology, The Second Hospital, Tianjin Medical University, Tianjin, China; 9 The Blood Purification Center, Tianjin People’s Hospital, TianJin, China; 10 Department of Nephrology, The Fourth Hospital, HeBei Medical University, ShiJia Zhuang, China; 11 Department of Nephrology, The First Hospital, Dalian Medical University, Dalian, China; 12 The Blood Purification Center, Beijing ChaoYang Hospital, Beijing, China; 13 Department of Nephrology, TongRen Hospital, Capital Medical University, Beijing, China; 14 Department of Nephrology, The Central Hospital of China Aerospace Corporation, Beijing, China; 15 Department of Nephrology, The Beijing Shiji Tan Hospital, Beijing, China; 16 Department of Nephrology, Dongzhi Men Hospital Affiliated to Beijing University of Chinese Medicine, Beijing, China; 17 Department of Nephrology, Beijing Hospital, Beijing, China; 18 Department of Nephrology, Xuanwu Hospital, Capital Medical University, Beijing, China

**Keywords:** Chronic kidney disease, Albuminuria, Renal function, Relatives, Spouses, Screening

## Abstract

**Background:**

Family members of patients with end stage renal disease were reported to have an increased prevalence of chronic kidney disease (CKD). However, studies differentiated genetic and non-genetic family members are limited. We sought to investigate the prevalence of CKD among fist-degree relatives and spouses of dialysis patients in China.

**Methods:**

Seventeen dialysis facilities from 4 cities of China including 1062 first-degree relatives and 450 spouses of dialysis patients were enrolled. Sex- and age- matched controls were randomly selected from a representative sample of general population in Beijing. CKD was defined as decreased estimated glomerular (eGFR < 60 mL/min/1.73 m^2^) or albuminuria.

**Results:**

The prevalence of eGFR less than 60 mL/min/1.73 m^2^, albuminuria and the overall prevalence of CKD in dialysis spouses were compared with their counterpart controls, which was 3.8% vs. 7.8% (P < 0.01), 16.8% vs. 14.6% (P = 0.29) and 18.4% vs. 19.8% (P = 0.61), respectively. The prevalence of eGFR less than 60 mL/min/1.73 m^2^, albuminuria and the overall prevalence of CKD in dialysis relatives were also compared with their counterpart controls, which was 1.5% vs. 2.4% (P = 0.12), 14.4% vs. 8.4% (P < 0.01) and 14.6% vs. 10.5% (P < 0.01), respectively. Multivariable Logistic regression analysis indicated that being spouses of dialysis patients is negatively associated with presence of low eGFR, and being relatives of dialysis patients is positively associated with presence of albuminuria.

**Conclusions:**

The association between being family members of dialysis patients and presence of CKD is different between first-degree relatives and spouses. The underlying mechanisms deserve further investigation.

## Background

Chronic kidney disease (CKD) is a global public health problem [1,2], and it affects 10-16% of the adult population in Asia, Australia, Europe and the United States [3,4]. A recent national survey in China [3] indicates that the prevalence of CKD in China is 10.8%, and the number of patients with CKD is estimated to 119.5 million. CKD has been associated with high morbidity and mortality [5], hence it is important to launch programs aiming at reducing the burden of CKD. It is reported that screening for proteinuria among high-risk population is cost-effective [6]. However, who constitute high-risk population for CKD remains to be answered.

Recent studies revealed that family members of patients with end stage renal disease (ESRD) have an increased prevalence of CKD [7-12]. Differences in ethnicities, lifestyles and screening methods may cause high variability in results [13,14]. Furthermore, some studies did not differentiate the genetic and non-genetic family members of patients with ESRD. A recent study from Taiwan revealed that both relatives and spouses of hemodialysis patients were found to have high prevalence of CKD [15]. The limited number of participants and the limited representativeness of controls constraint the power of that study. The present study was conducted to investigate the prevalence of CKD among the first-degree relatives and spouses of dialysis patients, and to compare that with controls from a representative sample of general population in Beijing.

## Methods

### Study population

Seventeen dialysis facilities from 4 cities of China were enrolled (12 in Beijing, 3 in Tianjin, 1 in Dalian and 1 in Shijiazhuang). ESRD Patients with inherited kidney disease, such as autosomal dominant polycystic kidney disease or Alport’s syndrome were excluded for this study. All family members, including first-degree relatives (including parent, sibling and child) and spouses of these patients were invited to participate in the study from October 2006 to August 2007. Altogether 1642 family members of ESRD patients participated in this study on a voluntary basis. Among them, 130 members who didn’t have either complete questionnaire or complete lab results were excluded. Finally, 1062 relatives and 450 spouses from 715 hemodialysis and 127 peritoneal dialysis families were eligible for present analysis. The ethics committee of Peking University First Hospital approved the study, which covers all participating institutions. All participants gave written informed consent before data collection.

Controls were selected from a representative sample of the general population of adults in Beijing, which is described in details elsewhere [16].

### Screening protocol and assessment criteria

Data were collected in examination centers at local health stations. All subjects completed a questionnaire documenting their sociodemographic status (e.g., age, sex, and educational level), health status (renal disease, diabetes mellitus, or hypertension), history of nephrotoxic medications (non-steroids anti-inflammatory drugs, [NSAIDS] or Chinese herbs containing aristolochic acid, [AA]), lifestyle behaviors (e.g., smoking), and the primary causes of renal failure of dialysis patients (glomerular disease, hypertension, diabetes, interstitial nephritis, and ‘all other’ causes).

Anthropometric measurements were obtained. Indicators of kidney damage and possible risk factors then were examined. All blood samples and urinary samples were tested in the central laboratory of Beijing University First Hospital.

### Definitions of CKD

Albumin and creatinine were measured from a fresh morning spot urine sample or morning urine sample stored at 4°C for less than 1 week. Albuminuria was measured using immunoturbidimetic methods (Audit Diagnostics, Cork, Ireland). Urinary creatinine was measured by means of Jaffe’s kinetic method on a Hitachi 7170 autoanalyzer (Hitachi, Tokyo, Japan). Urinary albumin-creatinine ratio (ACR; milligrams per gram) was calculated. Patients with ACR determinations that ranged from 17 to 250 mg/g (1.9 to 28.3 mg/mmol) for males and 25 to 355 mg/g (2.8 to 40.2 mg/mmol) for females were classified as having microalbuminuria, and participants with ACR values greater than the microalbuminuria range were classified as having macroalbuminuria. Albuminuria was defined as the presence of either microalbuminuria or macroalbuminuria. Women during menstruation were excluded from analyses for albuminuria.

Blood was collected by venipuncture after an overnight fast of at least 10 hours. Serum creatinine was measured by the same methods as was urinary creatinine. eGFR was calculated with an equation developed by modifying the Modification of Diet in Renal Disease (MDRD) equation based on data from Chinese CKD patients [17]. And decreased kidney function was defined as eGFR < 60 ml/min/1.73 m^2^ (1.00 ml/s/1.73 m^2^):

$$\begin{array}{l} \mathrm{eGFR}\;\left(\mathrm{ml}/\min/1.73m^{2}\right)\;=\;175\;\times \;\mathrm{Sc}r^{-1.234}\\ \;\times \;\mathrm{ag}e^{-0.179}\\ \;\times \;\left(\mathrm{if}\;\mathrm{female},\;\times \;0.79\right) \end{array}$$

where Scr is serum creatinine concentration (in mg/dL) and age in years.

The CKD was defined as decreased kidney function or albuminuria based on the classification system established by the National Kidney Foundation Kidney Disease Outcomes Quality Initiative (K/DOQI) [18].

### Definition of other conditions

Blood pressure was measured by sphygmomanometer, three times at 1 minute intervals. The mean of the three readings was calculated, unless the difference between readings was greater than 10 mmHg, in which case the mean of the two closet of the three measurements was used. Hypertension was defined as systolic blood pressure of 140 mmHg or greater or diastolic blood pressure of 90 mmHg or greater or use of antihypertensive medications in past 2 weeks irrespective of blood pressure, or any self-reported history of hypertension. Fasting blood glucose was measured enzymatically by means of a glucose oxidase method using the Hitachi 7170 autoanalyzer. Diabetes was defined as fasting plasma glucose of 7.0 mmol/L or more, by hypoglycaemic agents despite fasting plasma glucose, or any self-reported history of diabetes.

Serum total cholesterol, low-density lipoprotein cholesterol, high-density lipoprotein cholesterol, and triglycerides and uric acid were measured with commercially available reagents using a Hitachi 7170 autoanalyzer.

The body mass index (BMI) was calculated as weight (in kilograms) divided by height squared (in meters squared). Overweight is defined as BMI greater than 24 kg/m^2^. BMI = weight (kg)/height (m^2^) × 100%. Dyslipidemia was defined as present if total cholesterol was ≥ 5.72 mmol/L (220 mg/dL), or if low density lipoprotein (LDL) was ≥ 3.64 mmol/L (140 mg/dL), or triglyceride was ≥ 1.70 mmol/L (150 mg/dL) or high density lipoprotein (HDL) was < 0.91 mmol/L (35 mg/dL). Hyperuricaemia was defined as serum uric acid > 422 μmol/L for males and > 363 μmol/L for females.

### Statistics analysis

All analyses and calculations were performed by SPSS statistical package, version 16.0 (SPSS Inc., Chicago, IL, USA). Controls were selected from the cross-sectional survey of CKD in a representative sample of the general adults in Beijing [16]. Sample sizes of each age-stratified group of ≤ 30, 31–40, 41–50, 51–60, and > 60 years were 1814, 2816, 4206, 3002, and 2097 participants, respectively. In selecting controls for spouses, 900 sex- and age-stratified matched participants were randomly selected as controls. 2124 sex- and age-stratified matched participants were randomly selected as controls for relatives.

Data were presented as the mean ± standard deviation for continuous variables and as proportions for categorical variables. Descriptive analysis were used to characterize the participant population by sociodemographic data (eg. age, sex and education status) and health status (eg. hypertension and diabetes). Differences in variables between the two groups were analyzed using chi-square statistics for categorical variables or independent *t*-test for continuous variables. The unadjusted odds ratios (OR) between family members of dialysis patients and indicators of kidney damage were determined by univariate Logistic regression analysis. McNemar’s test was used to test univariate associations. A multivariate Logistic regression analysis was then performed to adjust for confoundings including age, gender, diabetes, hypertension, nephrotoxic medications, dyslipidemia, overweight, and chronic respiratory tract infection. Odds ratio (OR) was calculated and 95% confidence interval (CI) was provided. A *P* value of 0.05 or less was considered to be statistically significant.

## Results

### Spouses of dialysis patients

Characteristics of spouses and matched controls are listed in Table 1. eGFR was significantly higher in dialysis spouses than controls (86.9 ± 16.8 vs. 83.7 ± 18.1 mL/min/1.73 m^2^, P < 0.01). A significantly lower prevalence of low eGFR was found in dialysis spouses compared with controls (3.8% vs. 7.8%, P < 0.01). There were no differences in the prevalence of albuminuria (16.8% vs. 14.6%, P = 0.29) and CKD (18.4% vs. 19.8%, P = 0.61) between these two groups.

**Table 1:** Comparison of demographic and clinical characteristics between spouses of dialysis patients and controls

**Variables**	**Spouses (n = 450)**	**Controls (n = 900)**	**P Value**
Age (y)	57.9 ± 11.7	57.4 ± 12.6	0.51
Male (%)	181 (40.2)	362 (40.2)	1.0
Education (≥High School, %)	260 (57.8)	419 (46.7)	<0.001
Hypertension (%)	197 (43.9)	500 (55.7)	<0.001
Diabetes (%)	71 (15.8)	125 (14.0)	0.41
Overweight (%)	290 (64.7)	584 (64.9)	0.95
Nephrotoxic medications (%)	36 (8.0)	34 (3.9)	<0.01
Chronic respiratory tract infection (%)	106 (23.6)	158 (17.6)	0.01
Dyslipidemia (%)	231 (51.3)	378 (42.0)	<0.01
eGFR (mL/min/1.73 m^2^)	86.9 ± 16.8	83.7 ± 18.1	<0.01
Low eGFR (%) <60 (mL/min/1.73 m^2^)	17 (3.8)	70 (7.8)	<0.01
Albuminuria (%)	74 (16.8)	131 (14.6)	0.29
CKD (%)	83 (18.4)	178 (19.8)	0.61

Note: Data expressed as mean ± standard deviation or number (percentage); Overweight was defined as body mass index >24 kg/m^2^; Dyslipidemia was defined as present if total cholesterol was ≥ 5.72 mmol/L (220 mg/dL), or if low density lipoprotein was ≥ 3.64 mmol/L (140 mg/dL), or triglyceride was ≥ 1.70 mmol/L (150 mg/dL) or high density lipoprotein was < 0.91 mmol/L (35 mg/dL); Albuminuria was defined as urinary albumin-creatinine ratio ≥ 17 mg/g (1.9 mg/mmoL) for males and 25 mg/g (2.8 mg/mmoL) for females; CKD was defined as decreased kidney function (<60 mL/min/1.73 m^2^) or albuminuria.

Abbreviations: *eGFR*, estimated glomerular filtration rate; *CKD*, chronic kidney disease.

Among spouses, the prevalence of nephrotoxic medications use was higher and the prevalence of hypertension was lower compared with that of controls. There were no differences in prevalence of diabetes and overweight between these two groups. After adjusting for potential confounders, being spouses of dialysis patients was negatively associated with presence of decreased eGFR, with an OR of 0.50 (95% CI 0.28–0.88; Table 2).

**Table 2:** Unadjusted and adjusted odds ratio for indicators of kidney damage in the pooled database

**Variables**	Spouses (vs. controls)^*^	**Relatives (vs. controls)^†^**
Albuminuria		
Unadjusted OR (95% CI)	1.19 (0.87 – 1.62)	1.83 (1.45 – 2.31)
Age- and sex-adjusted OR (95% CI)	1.18 (0.86 – 1.62)	1.84 (1.45 – 2.32)
Multivariable adjusted OR (95% CI)	1.28 (0.92 – 1.77)^‡^	2.02 (1.57 – 2.59)^§^
Decreased eGFR		
Unadjusted OR (95% CI)	0.47 (0.27 – 0.80)	0.63 (0.36 – 1.11)
Age- and sex-adjusted OR (95% CI)	0.46 (0.27 – 0.80)	0.64 (0.36 – 1.13)
Multivariable adjusted OR (95% CI)	0.50 (0.28 – 0.88)^§^	0.68 (0.38 – 1.21) ^‡^
CKD		
Unadjusted OR (95% CI)	0.92 (0.69 – 1.23)	1.46 (1.17 – 1.82)
Age- and sex-adjusted OR (95% CI)	0.91 (0.67 – 1.22)	1.47 (1.17 – 1.83)
Multivariable adjusted OR (95% CI)	0.98 (0.72 – 1.33) ^‡^	1.58 (1.25 – 2.0)^§^

Abbreviations: *CKD*, chronic kidney disease; *OR*, odds ratio; estimated glomerular filtration rate; *CI*, confidence interval.

^*^ Pooled database including data of spouses and their counterpart controls.

^†^ Pooled database including data of relatives and their counterpart controls.

^‡^ORs were adjusted for age, gender, hypertension, diabetes.

^§^ORs were adjusted for age, gender, hypertension, diabetes, nephrotoxic medications, chronic respiratory tract infection, overweight, dyslipidemia, education (≥high school).

### First-degree relatives of dialysis patients

Characteristics of relatives and matched controls are listed in Table 3. A significantly higher prevalence of albuminuria (14.4% vs. 8.4%, P < 0.001) was found in relatives compared with controls. There was no difference in the prevalence of reduced eGFR between two groups (1.5% vs. 2.4%, P = 0.12).

**Table 3:** Comparison of demographic and clinical characteristics between relatives of dialysis patients and controls

**Variables**	**Relatives (n = 1062)**	**Controls (n = 2124)**	**P Value**
Age (y)	44.0 ± 12.1	43.7 ± 12.3	0.61
Male (%)	401 (37.8)	802 (37.8)	1.0
Education (≥High School, %)	810 (76.3)	1393 (65.8)	<0.001
Hypertension (%)	278 (26.3)	721 (34.0)	<0.001
Diabetes (%)	109 (10.3)	162 (7.7)	0.02
Overweight (%)	588 (55.6)	1182 (55.7)	1.0
Nephrotoxic medications (%)	67 (6.4)	51 (2.4)	<0.001
Chronic respiratory tract infection (%)	178 (16.9)	300 (14.1)	0.05
Dyslipidemia (%)	480 (45.2)	673 (31.7)	<0.001
eGFR (mL/min/1.73 m^2^)	94.0 ± 16.9	92.4 ± 18.9	0.03
Low eGFR (%) <60 (mL/min/1.73 m^2^)	16 (1.5)	50 (2.4)	0.12
Albuminuria (%)	149 (14.4)	178 (8.4)	<0.001
CKD (%)	155 (14.6)	221 (10.5)	<0.01

Note: Data expressed as mean ± standard deviation or number (percentage); Overweight was defined as body mass index >24 kg/m^2^; Dyslipidemia was defined as present if total cholesterol was ≥ 5.72 mmol/L (220 mg/dL), or if low density lipoprotein was ≥ 3.64 mmol/L (140 mg/dL), or triglyceride was ≥ 1.70 mmol/L (150 mg/dL) or high density lipoprotein was < 0.91 mmol/L (35 mg/dL); Albuminuria was defined as urinary albumin-creatinine ratio ≥ 17 mg/g (1.9 mg/mmoL) for males and 25 mg/g (2.8 mg/mmoL) for females; CKD was defined as decreased kidney function (<60 mL/min/1.73 m^2^) or albuminuria.

Abbreviations: *eGFR*, estimated glomerular filtration rate; *CKD*, chronic kidney disease.

The prevalence of diabetes and nephrotoxic medication use were higher among relatives compared with those of controls, while the prevalence of hypertension was lower among relatives compared with that of controls. After adjusting for potential confounders, being relatives of dialysis patients was positively associated with presence of albuminuria, with an OR of 2.02 (95%CI 1.57–2.59; Table 2).

## Discussion

Our study revealed a higher prevalence of albuminuria among first-degree relatives of dialysis patients, and the positive association is independent of various potential confounders. Furthermore, we observed a lower prevalence of decreased renal function among spouses of dialysis patients compared with controls. A major strength of our study is the large sample size and the representativeness of controls.

The high prevalence of CKD among first-degree relatives of dialysis patients in our study is consistent with previous studies [19,20]. Compared with previous screening programs for high-risk populations, the prevalence of albuminuria in relatives of dialysis patients (14.4%) in this study was lower than that from the Kidney Early Evaluation Program (KEEP) study (29%) [21], but similar to that from Tsai’s study (10.7%) [15]. This figure was higher than the observed rate of albuminuria in a general population-based screening program in China (9.4%)^3^ and in Europe and the United States (5.9% – 7.4%) [22,23]. One possible explanations might be familial clustering of metabolic disorders, such as hypertension and diabetes, which are known risk factors of albuminuria. Previous studies suggested that, of these renal risk factors, hypertension and diabetes mellitus are multifactorial disease under the influence of both genetic traits and environmental factors [10,24]. Environmental factors, such as low socioeconomic status and lifestyle of inactivity and smoking, also should be considered as potential contributing for CKD [25,26]. However, in our analyses, after adjusting for potential confounders, the positive association between being relatives of dialysis patients and albuminuria still exist, indicating that there might be genetic susceptibility of CKD for relatives.

Genetic traits may contribute to the development of CKD in relatives of dialysis patients. It is well known that familial focal segmental glomerulosclerosis (FSGS) is a significant and growing cause of CKD. Given the progress in understanding the biology and pathology of podocyte, mutation of associated genes, such as ACTN4, TRPC6 and NPHS2, contribute to the damage of podocyte and podocyte dysfunction [27,28]. The latter was associated to the development of proteinuria and FSGS [29]. These genes were recognized to be the genetic basis of FSGS. Meanwhile, more related genes or chromosomal regions were identified in diabetic (3q, 18q22.3-23), non-diabetic nephropathy (chromosome 10), systemic lupus erythematosus and familial IgA nephropathy (6q22-23) [30-32].

Spouses of patients with ESRD were considered to be nongenetic controls for studying the family clustering of CKD [11]. Spousal concordance of health risks and behaviors such as cardiovascular disease, hypertension, metabolic syndrome and high fasting glucose levels has been observed in many disease [33-35]. In this study, we also found higher rates of some health risks in dialysis spouses compared with controls, such as dyslipidemia and use of nephrotoxic medications. However, this study demonstrated a significantly lower prevalence of low eGFR, but no differences in the prevalence of albuminuria and CKD in dialysis spouses compared with controls. Meanwhile, in our analyses, after adjusting for potential confounders, the negative association between being spouses of dialysis patients and low eGFR still exist. These results were enhanced by the sufficient number of participants from multicentric facilities and the ample representativeness of sex- and age- matched controls, which were randomly selected on a ratio of 2:1 from a representative sample of the general population of adults in Beijing [16]. The exact reason was unknown. We assumed that for being spouses of dialysis patients, perceived and objective CKD knowledge are likely to impact risk-modifying behavior in different ways. Multi-component structured empowerment intervention is effective in pre-dialysis CKD patients and may lead to a delay in the progression of kidney disease [36-40].

Our study has limitations that deserve mention. Firstly, it was implemented on a voluntary bias within the dialysis units. Additionally, ESRD patients who didn’t start dialysis treatment were not included in present study. There were kinds of selecting bias in the study which limited the extension of the results from this study. Secondly, urine and blood results were based on a single measurement. It should be noticed that it might overestimate the prevalence of albuminuria based on one measurement. Finally, a cross-sectional study design has several inherent weaknesses, such as lack of long-term observation for outcome and difficulty interpreting the association of exposure with outcome.

## Conclusions

In conclusion, the association between being family members of dialysis patients and presence of CKD is different between first-degree relatives and spouses. Genetic susceptibility may account for the phenomenon of family clustering of CKD. However, non-genetic environmental factors also should be considered as potential contributing for CKD. The underlying mechanisms deserve further investigation. Strategies aimed at intervention of hypertension and other metabolic disorders might prove effective in controlling the pandemic of CKD in family members of dialysis patients.

## Competing interests

The authors declare that they have no competing interests.

## Authors’ contributions

KX and ZL participated in the study, analyzed the data, interpreted the results, and drafted the manuscript. LL, ZL, YP, LZ, LW, CM, CX, JA, LG, XJ, LH, WS, HW, WY, GY, CP, WH and JQ participated in the survey and study design and collected the data. ZL and WM formed the study concept, interpreted the results, and revised the manuscript. WH revised the manuscript for important intellectual content. All authors read and approved the final manuscript.

## Pre-publication history

The pre-publication history for this paper can be accessed here:

http://www.biomedcentral.com/1471-2369/14/19/prepub

## Support

This work was funded by the development and validation of new markers for diagnosis and evaluation of CKD (09050704310902) from the Beijing Science and Technology Committee and the grants for the Early Detection and Prevention of Non-communicable Chronic Diseases from the International Society of Nephrology Research Committee, and the grants from the China Health and Medical Development Foundation.
